# Double-edged mitophagy: balancing inflammation and resolution in lung disease

**DOI:** 10.1042/CS20256705

**Published:** 2025-09-30

**Authors:** Sijia Tian, Yingyi Zhang, Chuanchuan Liu, Huajing Zhang, Qianying Lu, Yanmei Zhao, Haojun Fan

**Affiliations:** 1 School of Disaster and Emergency Medicine, Tianjin University, 300072, China; 2 Tianjin Key Laboratory of Disaster Medicine Technology, Tianjin, 300072, China

**Keywords:** inflammation, lung diseases, mitophagy, mtROS, therapeutic targeting

## Abstract

Inflammatory lung diseases, such as chronic obstructive pulmonary disease (COPD), acute lung injury (ALI)/acute respiratory distress syndrome (ARDS), and asthma, are driven by mitochondrial dysfunction and aberrant immune responses, yet the regulatory role of mitophagy—a selective autophagy eliminating damaged mitochondria—remains poorly defined. This review synthesizes evidence from *in vivo* and *in vitro* studies to dissect the molecular interplay between mitophagy and inflammation. Key fundings reveal that mitophagy exerts context-dependent effects: Protective mitophagy (via PTEN-induced putative kinase 1 [PINK1]-Parkin or FUN14 domain-containing protein 1 [FUNDC1] pathways) clears mitochondrial reactive oxygen species (mtROS)/mitochondrial DNA (mtDNA), suppressing NOD-like receptor thermal protein domain associated protein 3 (NLRP3) inflammasome activation and pyroptosis, but excessive mitophagy exacerbates mitochondrial fragmentation and necroptosis. Notably, bidirectional cross-talk exists, and therapeutic strategies—genetic and pharmacological—could restore mitophagy flux, attenuating inflammation in preclinical models. However, challenges persist in targeting tissue-specific mitophagy (such as alveolar and bronchial epithelia). This work underscores mitophagy as a double-edged sword in lung inflammation and proposes precision interventions to balance mitochondrial quality control, offering novel avenues for inflammatory lung diseases.

## Introduction

As the main organ in the body responsible for gas exchange, the lungs are directly exposed to a wide array of environmental pollutants. Consequently, they are vulnerable to both external environmental factors and internal influences, which can easily lead to injury and progressive deterioration, ultimately impairing quality of life and potentially leading to death [1–3]. Inflammatory lung diseases, such as acute lung injury/acute respiratory distress syndrome (ALI/ARDS), chronic obstructive pulmonary disease (COPD), asthma, and airway inflammation, represent prevalent public health challenges that impose substantial burden on healthcare systems [4]. The World Health Organization reported that, as of 2021, COPD has become the third leading cause of death worldwide [5]. Therefore, it is crucial to investigate innovative treatment approaches for inflammatory lung conditions [6]. While acute or chronic inflammation is a hallmark of these conditions, conventional therapies targeting immune cells, such as corticosteroids and cytokine inhibitors, often yield suboptimal outcomes due to their inability to address the root cause: mitochondrial dysfunction [7].

Mitochondria are the main hubs for aerobic respiration and adenosine triphosphate (ATP) production within cells [8]. When damaged, they release mitochondrial damage-associated molecular patterns (mtDAMPs), such as mitochondrial DNA (mtDNA) and mitochondrial reactive oxygen species (mtROS), which activate pattern recognition receptors like NOD-like receptor thermal protein domain associated protein 3 (NLRP3) and toll-like receptor 9 (TLR9), fueling a vicious cycle of inflammation and tissue injury [9]. To mitigate this, cells employ mitophagy, a selective autophagy mechanism that identifies and removes dysfunctional mitochondria [10]. Pioneering studies by Lemasters and Youle revealed PTEN-induced putative kinase 1 (PINK1)-Parkin pathway as a central regulator of mitophagy [11], yet emerging evidence suggests intricate cross-talk between ubiquitin-dependent and independent pathways in maintaining mitochondrial homeostasis.

Despite progress, critical knowledge gaps persist. First, the dual role of mitophagy in lung diseases remains paradoxical: while adequate mitophagy suppresses NLRP3 inflammasome activation (such as PINK1-mediated mtROS clearance in sepsis), excessive removal of mitochondria (as seen in hyperactive Parkin models) depletes cellular energy reserves, exacerbating epithelial apoptosis. Second, the disease specificity of mitophagy pathways is poorly characterized. For instance, FUN14 domain-containing protein 1 (FUNDC1) dominates hypoxia-induced mitophagy in pulmonary fibrosis (PF), whereas BCL2/Adenovirus E1B 19 kDa protein-interacting protein 3 (BNIP3) is pivotal in asthma-related airway remodeling. Third, current therapeutic strategies—genetic (such as silent information regulator 3 [SIRT3] overexpression) or pharmacological (such as melatonin)—lack precision in targeting spatially distinct mitochondrial populations (such as alveolar and bronchial epithelia).

This review seeks to bridge these gaps by addressing three pivotal questions. How do divergent mitophagy pathways modulate inflammatory responses in specific lung diseases? What are the bidirectional interactions between mitophagy and key inflammatory signaling axes (NLRP3, cyclic GMP-AMP synthase [cGAS]-stimulator of interferon genes [STING], nuclear factor-κB [NF-κB])? Can we harness tissue-specific mitophagy regulation to develop safer therapeutics, particularly integrating traditional Chinese medicine (TCM) with molecular interventions?

By synthesizing data from preclinical models and clinical cohorts, we unveil mitophagy as a context-dependent rheostat of lung inflammation. Our analysis highlights that restoring mitochondrial quality control—rather than uniformly enhancing or inhibiting mitophagy—may hold the key to breaking the inflammatory cascade. These insights not only refine our understanding of lung pathophysiology but also pave the way for personalized therapies combining mitochondrial precision medicine with holistic approaches.

## Mitochondrial homeostasis and mitophagy pathways

**Figure 1:**
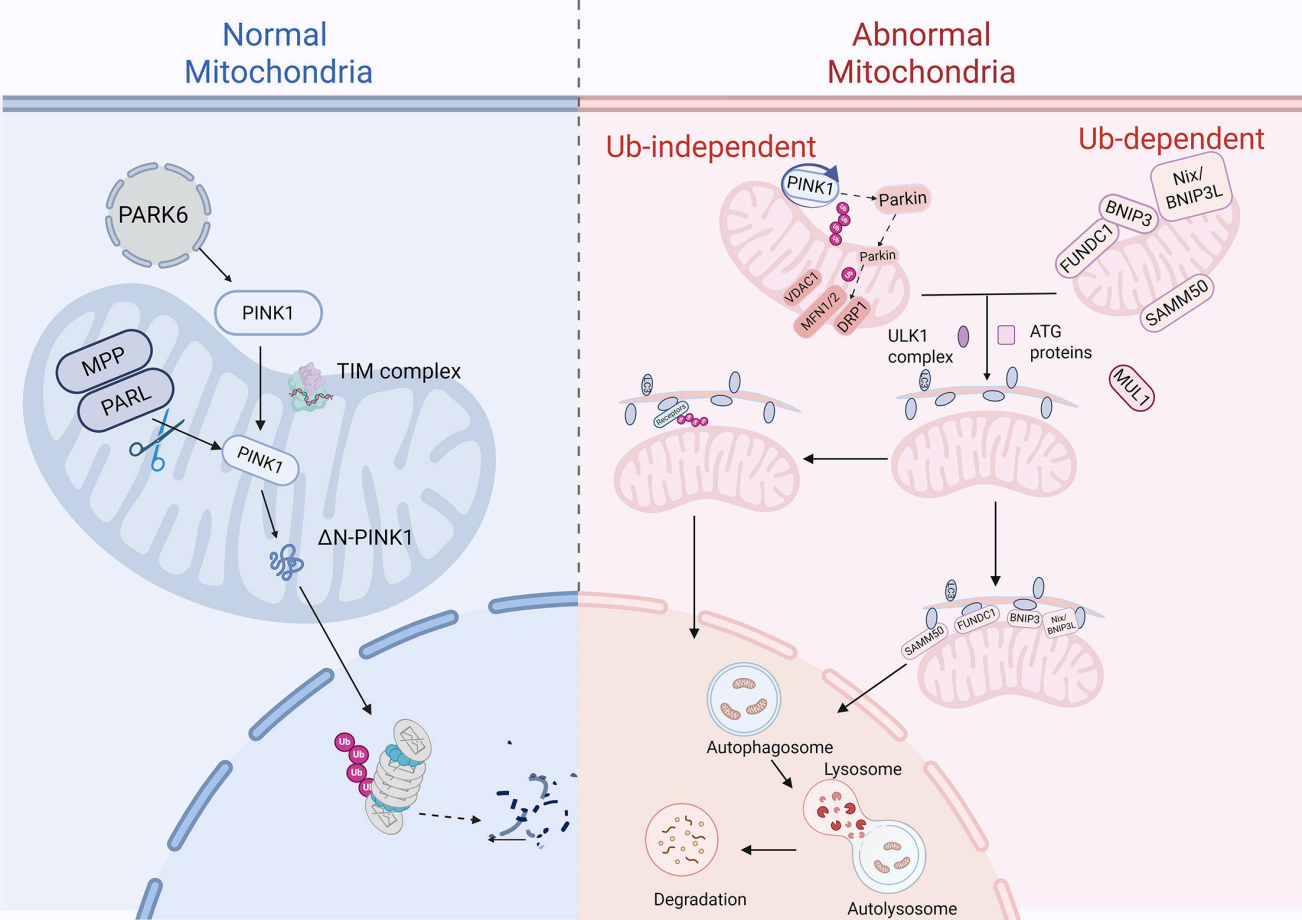
Mitophagy pathways. In normal mitochondria, PARK6 gene coding transports PINK1 into the mitochondria via the TIM complex, where PINK1 is cleaved by MPP and PARL into ΔN-PINK1, maintaining mitochondrial function. In abnormal mitochondria, there are two autophagy mechanisms: ubiquitin-independent and ubiquitin-dependent. In the ubiquitin-independent mechanism, PINK1 accumulates and recruits Parkin protein to initiate the autophagy process. In the ubiquitin-dependent mechanism, proteins such as Nix/BNIP3L participate in ubiquitination marking, promoting the formation of autophagosomes. Ultimately, abnormal mitochondria are degraded through the fusion of autophagosomes with lysosomes. MPP, mitochondria-penetrating peptide; PARK6/PINK1, PTEN-induced putative kinase 1; PARL, progerin-associated rhodopsin; TIM, translocase of inner mitochondrial membrane. Created in Biorender.u0. (2025) https://BioRender.com/aoyjyr5.

Mitochondrial homeostasis hinges on the precise balance between biogenesis, gynamics, and degradation. Central to this equilibrium is mitophagy, a selective autophagy mechanism that eliminates dysfunctional mitochondria to prevent the accumulation of cytotoxic mtDAMPs. Two evolutionarily conserved pathways govern mitophagy: ubiquitin-dependent and ubiquitin-independent mechanisms, each responding to distinct stress signals [12] (Figure 1).

### Ubiquitin-dependent pathways

The ubiquitin-dependent pathway plays a crucial role in mitophagy by heavily ubiquitinating the proteins on mitochondrial surfaces. This extensive ubiquitination leads to the formation of ubiquitin chains on mitochondria, which is a vital step in the mitophagy process, as it helps determine which mitochondria are marked for degradation and removal via autophagy. The most thoroughly researched aspect of this pathway is the PINK1-Parkin mechanism, characterized by three key components: PINK1 acts as a sensor for mitochondrial damage, Parkin serves as a signal enhancer, and the ubiquitin chains function as the signaling effectors [13]. Together, these proteins orchestrate the activation of mitophagy in response to mitochondrial damage.

PINK1 is a mitochondrial protein encoded by the type 6 of Parkinson’s disease gene [14] with an N-terminal mitochondrial-targeting sequence [15], enabling its specific translocation to the inner mitochondrial membrane (IMM). Under homeostatic conditions, PINK1 is imported into healthy mitochondria and rapidly cleaved by progerin-associated rhodopsin, maintaining low baseline levels [16,17]. When mitochondria are damaged, the mitochondrial membrane potential (MMP/ΔΨm) alters, hindering the movement of PINK1 to the IMM, triggering its accumulation on the outer mitochondrial membrane (OMM). This activated form of PINK1 subsequently attracts and activates Parkin, an E3 ubiquitin ligase, which ubiquitinates OMM proteins, such as mitofusin1/2 (MFN1/2), voltage-dependent anion channel 1, to form ‘eat-me’ signals. These polyubiquitin chains are recognized by autophagy receptors, linking tagged mitochondria to microtubule-associated protein 1 light chain 3 (LC3)-labeled autophagosomes [18].

Interestingly, PINK1 can bypass Parkin under certain conditions. For example, PINK1 can promote Parkin-independent mitophagy by directly attracting optineurin (OPTN) and nuclear dot protein 52 kDa (NDP52) to the mitochondria through a process involving ubiquitination and phosphorylation [19]. Furthermore, various other E3 ubiquitin ligases participate in mitophagy, including seven in absentia homolog-1 (SIAH1) and mitochondrial E3 ubiquitin protein ligase 1 (MUL1). SIAH1 is a ring-type E3 ubiquitin ligase that collaborates with PINK1 and synphilin-1 to form the PINK1-synphilin-1-SIAH-1 complex. This complex facilitates the recruitment of LC3 and the lysosomal marker lysosomal associated membrane protein 1 (LAMP1) to the mitochondria, effectively kickstarting the mitophagy process [20]. MUL1 deficiency could increase MFN2 activity, induce mitochondrial over-fusion, and impair endoplasmic reticulum and mitochondria (ER-Mito) interactions. This disruption negatively affects mitochondrial bioenergetics and calcium homeostasis, ultimately triggering mitochondrial fragmentation and Parkin-mediated mitophagy through the activation of calmodulin phosphatase and dynamin-related protein 1 (DRP1) [21].

### Ubiquitin-independent pathways

In addition to the classical PINK1-Parkin pathway, several other mitophagy receptors have been identified on the OMM that can initiate mitophagy by directly binding to LC3 via LC3-interacting regions without ubiquitination, which gives rise to a ubiquitin-independent mitophagy pathway.

#### Hypoxia-responsive receptors

BNIP3, BNIP3L, and FUNDC1 are three key regulatory proteins of mitophagy. In addition to regulating mitophagy individually, they collectively respond to hypoxic conditions and participate in the mitophagy. First, Nix/BNIP3L and BNIP3, as homologous proteins, are regulated by hypoxia-inducible factor-1α (HIF-1α) under hypoxic conditions [22]. HIF-1α can up-regulate the transcription levels of BNIP3 and BNIP3L, making them displace Bcl-2 from Beclin-1 (BECN-1), enhancing autophagosome formation while inducing mitochondrial clearance [23,24]. The mitophagy process driven by FUNDC1 in low-oxygen conditions relies on the collaboration between the ER and mitochondria. Under hypoxic circumstances, FUNDC1 associates with calnexin, a membrane protein found within the ER, and facilitates mitochondrial division and attracts UNC-51 like kinase to mitochondria-ER contact (MERC) sites, coupling mitochondrial fission with autophagosome assembly [25,26].

#### Lipid-mediated recognition

In addition to protein receptors, lipids can bind LC3 and act as autophagy receptors. Cardiolipin (CL), a signature phospholipid found almost exclusively in the IMM, responds to a variety of mitochondrial stresses thereby translocating to the OMM [27]. When mitochondria are disrupted by carbonyl cyanide 3-chlorophenylhydrazone (CCCP), CL binds to the hexameric membrane spacer protein NDPK-D (NM23-H4), which is transferred from the IMM to the OMM, where it accumulates [28]. When the amount of CL on the OMM is increased, it is recognized by LC3A/B, which promotes mitophagy [29]. In addition, the CL of OMM can directly interact with BECN-1 to participate in mitophagy [30]. Ceramides, particularly C18-ceramide, are produced through the action of ceramide synthase 1 (CERS1) catalysis and localized to the OMM [31]. There is a notable structural resemblance between the globular domain of LC3B and the ceramide-binding domain of ceramide transporter proteins. This similarity facilitates the interaction between LC3B and C18-ceramide, enhancing the recruitment and proliferation of autophagosomes, which in turn triggers the process of mitophagy [31].

The choice between ubiquitin-dependent and independent pathways is dictated by stress type and cellular context. For example, PINK1-Parkin dominates in toxin-induced mitochondrial damage (such as rotenone), whereas FUNDC1 is critical in ischemia-reperfusion injury. Dysregulation of this selectivity underlies disease pathogenesis: impaired PINK1-Parkin signaling exacerbates mtDAMPs release in COPD, while excessive BNIP3 activation drives fibrosis via unresolved oxidative stress. This compartmentalized yet interconnected regulation underscores mitophagy’s dual role as a guardian of homeostasis and a potential instigator of pathology.

## Mitophagy dysregulation in inflammatory lung diseases

**Figure 2:**
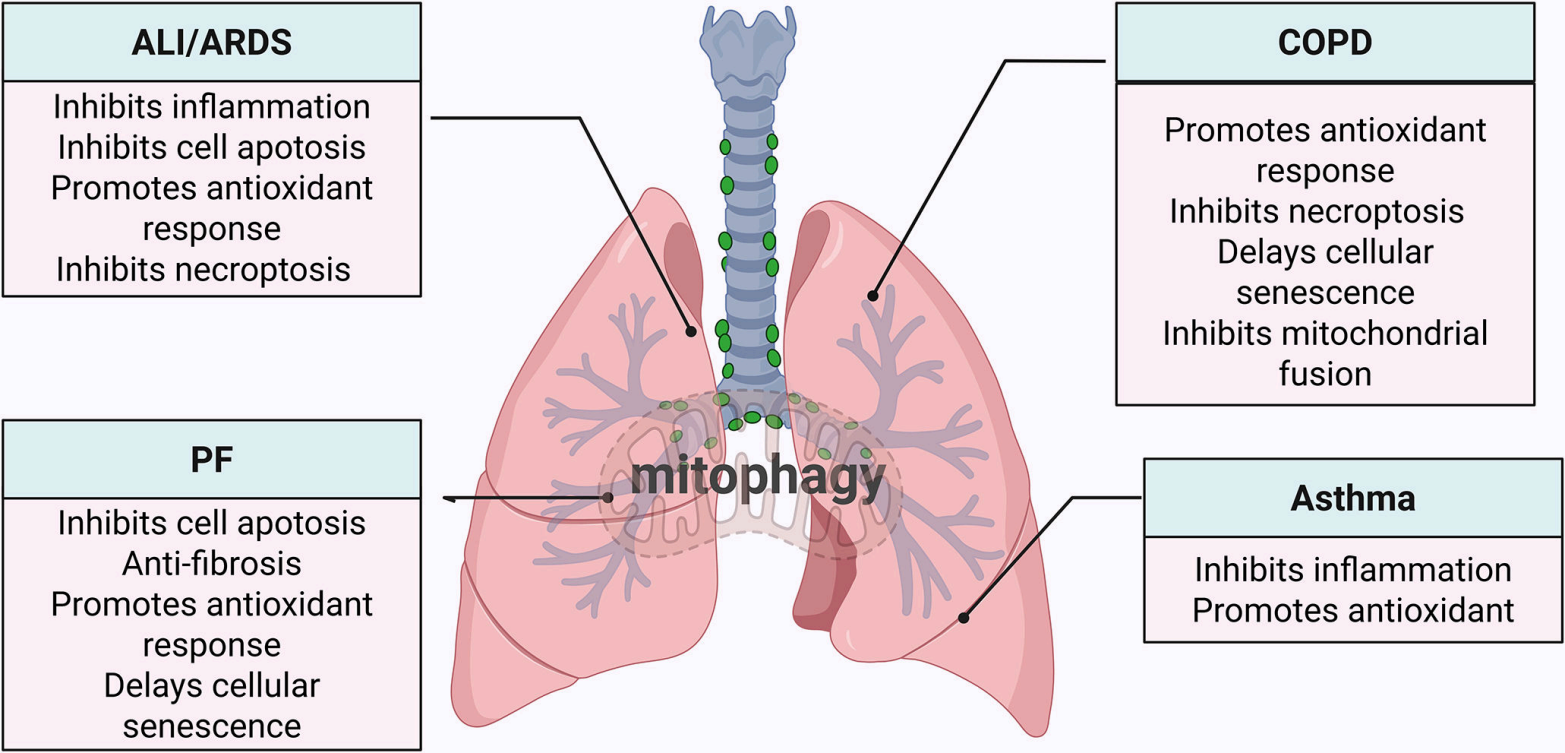
Role of mitophagy in different inflammatory lung diseases. It shows the multiple roles of mitophagy in these diseases, such as inhibiting inflammation, apoptosis, and necroptosis, as well as promoting antioxidant responses and anti-fibrotic effects. Mitophagy has also been found to delay cellular senescence, inhibit mitochondrial fusion. ALI/ARDS, acute lung injury/acute respiratory distress syndrome; COPD, chronic obstructive pulmonary disease; PF, pulmonary fibrosis. Created in biorender.u0. (2025) https://BioRender.com/wdfcrvg.

Mitophagy, serving as a critical balancer of mitochondrial quality and quantity within lung cells, plays an essential role in maintaining pulmonary homeostasis. This selective form of autophagy is essential for clearing damaged or dysfunctional mitochondria, as their buildup can result in heightened ROS production, energy deficits, and the escalation of inflammatory signals. Thus, the careful regulation of mitophagy is key to upholding cellular integrity and modulating lung inflammation. When mitophagy is disrupted, it is linked to various inflammatory lung diseases, such as ALI/ARDS, COPD, PF, asthma, and other inflammatory conditions. In this section, we have investigated the role of mitophagy in lung disorders, aiming to offer novel insights into the pathogenic mechanisms and therapeutic approaches for lung conditions (Figure 2).

### Acute lung injury/acute respiratory distress syndrome

ALI-ARDS is a common and serious clinical syndrome characterized by a high incidence rate [32]. Despite advancements in treatment and nursing practices in recent years, the mortality rate continues to be significant, ranging from 30% to 40%, due to the intricate pathophysiological mechanisms involved. Its main pathological feature is diffuse damage to alveolar epithelial cells (AEC) and pulmonary capillary endothelial cells [33].

Research conducted *in vitro* and *in vivo* has shown that mtROS is pivotal in regulating the endothelial barrier and lung injury [34]. Accumulation of ROS within cells during episodes of lung injury leads to the activation of inflammatory mediators like mitogen‐activated protein kinases (MAPKs) and NF-κB complexes [35]. This cascade of events ultimately compromises the integrity of the epithelial barrier, resulting in the leakage of inflammatory substances and exacerbating ALI [36]. Mitophagy has been shown to eliminate damaged mitochondria and decrease ROS production, thus mitigating ALI [37]. Therefore, the level and impact of mitophagy are the key points of research, and insufficient or excessive mitophagy will have an impact on the process of ALI-ARDS.

Initially, inadequate mitophagy results in the buildup of impaired mitochondria, which damage cellular energy metabolism and promote apoptosis, exacerbating pulmonary inflammation and injury. Lipopolysaccharide (LPS)-induced ALI models show impaired PINK1-Parkin signaling due to sestrin2 (Sens2) or B-cell receptor-associated protein 31 (BCAP31) deficiency, exacerbating lung injury [38]. Sens2 induces autophagy by activating AMP-dependent protein kinase (AMPK) and inhibiting mechanistic target of rapamycin complex 1 (mTORC1) signaling, which has a protective effect against ALI [39]. BCAP31, an essential element of the MAM, plays a crucial role in the transport and positioning of mitochondrial proteins, thereby ensuring the stability of mitochondrial function [40]. The stimulation of the PINK1-Parkin pathway has been shown to mitigate LPS-induced mitochondrial injury and cell death, indicating that BCAP31 serves as a beneficial modulator of mitophagy, opening up promising avenues for addressing ALI [41,42].

However, excessive mitophagy, as noted in studies, can also be detrimental, potentially leading to reduced ATP production, a shortfall in cellular energy, and increased ROS levels. This overactivation can also impair ΔΨm and disrupt the electron transport chain, thereby exacerbating the development process of ALI. In mice with LPS-induced ALI, Zhang’s team discovered an excessive increase in mitophagy and revealed for the first time the critical role of Bcl-2 and Bad in regulating mitophagy in ALI. Bcl-2 family proteins are considered to be key regulators of apoptosis, acting as anti-apoptotic proteins by regulating the permeability of the OMM to different proteins [43]. At the MERC site, Bcl-2 protein family can dynamically control Ca^2+^ through direct interactions with intracellular Ca^2+^ transporters or channels to maintain mitochondrial homeostasis [44]. Specifically, LPS stimulation has been observed to decrease Bcl-2 expression and increase Bad expression. As the ΔΨm dissipates, the PINK1-Parkin pathway is activated, initiating mitophagy. Bcl-2 directly interacts with Parkin to inhibit its recruitment from the cytoplasm to the mitochondria, while Bad directly suppresses the anti-mitophagy protein Bcl-2. Consequently, overexpression of Bcl-2 and knockdown of Bad both reduce mitophagy, thereby alleviating LPS-induced ALI in mice [45]. These findings revealed that LPS shifts the Bcl-2-Bad equilibrium, hyperactivating Parkin-mediated mitophagy and mitochondrial fragmentation. This paradox is further highlighted by Parkin’s dual role: while its overexpression alleviates injury in heat stroke models, chronic LPS exposure paradoxically suppresses Parkin, suggesting a self-protective feedback to prevent mitochondrial over-clearance.

Notably, FUNDC1-driven mitophagy in AECs links citrate accumulation to DRP1-dependent fission, bridging metabolic stress to necroptosis via triggering receptor expressed on myeloid cells 1 (TREM-1)-mammalian target of rapamycin (mTOR) signaling. Cell apoptosis represents a genetically orchestrated mechanism of cellular demise characterized by the activation of caspase enzymes and unfolds through a systematic series of events. On the other hand, necrosis has traditionally been viewed as cell death resulting from severe physical or chemical trauma. Nevertheless, recent studies have unveiled a novel type of necrosis known as necroptosis, which is now acknowledged as a significant contributor to apoptotic epithelial cell death in the context of ALI [46,47]. Yang’s team identified a novel mechanism of necroptosis in which the accumulation of mitochondrial citrate leads to necroptosis in AEC exposed to LPS [48]. This process is associated with the down-regulation of Idh3α and citrate carrier (CIC), which are crucial for maintaining citrate homeostasis. The interaction of MT with FUNDC1 enhances FUNDC1’s binding to DRP1, triggering mitochondrial fission and increased mitophagy. This chain of events ultimately leads to necroptosis and contributes to the progression of ALI [48]. The activation of TREM-1 has been shown to stimulate the p-DRP1 at Ser616 through the mTOR pathway, which in turn induces excessive mitochondrial fission and macrophage necroptosis, contributing to the severity of ALI [49].

### Chronic obstructive pulmonary disease

COPD, a prevalent chronic respiratory disorder, is defined by persistent airflow obstruction, often resulting from exposure to inhaled particulates like cigarette smoke, environmental infections, air pollution, and other etiological factors [50,51]. According to data from 2019, COPD is globally ranked as the third leading cause of mortality. Studies have shown that reduced ΔΨm [52], impaired mitochondrial respiratory chain function, and enhanced ROS production are shown in COPD, suggesting that mitophagy may be involved in COPD [53].

Elevated levels of PINK1 and decreased levels of Parkin are associated with the pathogenesis of COPD related to mitophagy [54]. However, research has revealed that overexpression of Parkin can trigger mitophagy during exposure to cigarette smoke (CS) in the context of decreased PINK1 levels. Conversely, when PINK1 is overexpressed, it is unable to repair the damaged mitophagy caused by reduced Parkin levels. This suggests that the level of Parkin protein may be a key limiting factor in the PINK1-Parkin-mediated mitophagy process during cigarette smoke extract (CSE) exposure, indicating that Parkin induction may help mitigate the progression of COPD.

Mitophagy may influence COPD progression by modulating necroptosis. Mizumura and colleagues, using a COPD model induced by CSE in lung ECs, found that CSE triggers the expression of PINK1 and the phosphorylation of DRP1 [55]. This induces PINK1-dependent mitophagy, leading to a decrease in ΔΨm and resulting in the death of lung ECs. In vivo studies also revealed that mitochondrial integrity was compromised, and the expression of LC3B and RIP3 increased after three months of CSE exposure, suggesting that an enhanced mitophagy process can significantly damage mitochondria and induce necroptosis. However, when PINK1 expression was knocked down or cells were treated with Mdivi-1, a mitochondrial fission inhibitor, the phosphorylation of mixed lineage kinase domain-like protein induced by CSE was reduced. Moreover, the necroptosis inhibitor Nec-1 had no effect on CSE-induced PINK1 expression, further confirming that PINK1-dependent mitophagy may act as an upstream regulator of the necroptosis pathway. These findings provide a new perspective on the pathogenesis of COPD, suggesting that modulating PINK1-dependent mitophagy could alleviate the disease by inhibiting the process, thereby reducing the mitochondrial damage and necroptosis that exacerbate COPD.

In addition, mitophagy participates in the process of COPD by regulating cellular senescence [54]. In the CSE-induced COPD model of mice, CSE exposure induces deficits in MFN2 and optic atrophy 1 (OPA1), which causes mitochondrial dysfunction and cellular senescence. Studies have shown that knocking down OPA1 or MFN2 increases mtROS production and the percentage of senescent cells in human bronchial epithelial cells [56]. Mitochondrial fragmentation, autophagosome equivalence, and aberrant expression of mitochondria-associated proteins including decreased levels of OPA1 and PINK1, and elevated levels of the mobilization of protein sequestosome-1 (SQSTM1/p62) and LC3B II-LC3B I were observed in lung tissues and non-cellular cells from COPD patients. Concurrently, the expression of senescence-associated proteins P16 and histone H2A.X increases, while the expression of the anti-aging protein Klotho decreases. These changes collectively promote the aging process of lung cells. By using drug inducers or genetically inducing the expression of MFN2 and OPA1, it is possible to improve mitochondrial morphology and function, reduce mtROS, lower senescence-associated secretory phenotype mRNA levels, decrease the expression of aging-related proteins, and enhance the oxidative phosphorylation system capacity of cells. Furthermore, it has reported that CSE exposure alters the abundance of key mitochondrial proteins such as PINK1 and Parkin, and chronic CSE exposure leads to excessive mitophagy, leading to the mitochondria accumulating around the nuclei of lung epithelial and fibroblast cells. Overexpression of Parkin restored mitophagy and mitigated the accumulation of mitochondrial quality in CSE-treated cells, significantly reducing mtROS and cellular senescence, with mtROS being a key inducer of aging. Therefore, by modulating the expression of mitochondrial fusion proteins such as MFN2 and OPA1, it is possible to improve mitochondrial function, promote mitophagy, alleviate oxidative stress and cellular senescence, and thus ameliorate the progression of COPD [57].

### Pulmonary fibrosis

PF is a persistent and often fatal lung condition, with various known triggers such as certain medications and autoimmune or connective tissue disorders [58]. Among the different forms of PF, idiopathic pulmonary fibrosis (IPF) stands out as the most aggressive variant, marked by a relentless progression of fibrosis [59]. This condition is distinguished by its irregular fibrotic patterns both temporally and spatially, the occurrence of fibroblastic foci, and an unstructured and excessive accumulation of collagen and extracellular matrix [60,61]. These alterations ultimately lead to the loss of normal lung architecture, often accompanied by the formation of honeycomb cysts. Together, these symptoms and pathological changes propel the progression of PF, causing severe impairment of lung function. In the pathogenesis of IPF, the susceptibility of alveolar epithelial type II (AECII) to ER stress is a widely accepted theory [62]. AECII contains approximately 50% of the lung’s mitochondrial mass, and mitochondrial structure and function are regulated by ER stress and autophagy processes. Studies indicate that PINK1-deficient mice develop deformed, dysfunctional mitochondria in AECII and are prone to apoptosis, which promotes the progression of PF. Therefore, AECII damage and defects in mitophagy may be key factors in the development of IPF [63].

In PF, defective mitophagy perpetuates fibroblast activation through mtROS-NLRP3-IL-1β axis overactivation. PINK1-deficient mice exhibit distorted AECII mitochondria and accelerated apoptosis, priming fibrotic remodeling. Conversely, thymosin β4 (Tβ4) enhances PINK1-TOM40 mitophagy, dampening oxidative damage and collagen deposition. The TGF-β1-Akt1 axis further suppresses mitophagy in alveolar macrophages, amplifying mtROS and pro-fibrotic signaling.

In PF, mitophagy plays a crucial role in inhibiting cellular senescence. BMP4, a member of the TGF-β1 superfamily and a pleiotropic cytokine, orchestrates cellular proliferation and differentiation in embryonic development, was down-regulated and negatively correlated with fibrotic genes in IPF patients and a bleomycin-induced PF mouse model [64]. Insufficiency in mitophagy leads to excessive ROS production, inducing senescence in fibroblasts and accelerated differentiation into myofibroblasts. Elevated levels of BMP4 lead to a reduction in PINK1 expression, which in turn lessens the down-regulation of Beclin-1 and LC3II, along with the increased levels of p62 caused by TGF-β1. This chain of events helps to alleviate the impairments associated with TGF-β1-stimulated mitophagy and the excessive generation of intracellular mtROS. Moreover, BMP4 lowers the expression of p53, a key tumor suppressor gene, and p21, which contributes to a slowdown in cellular aging [65]. These findings suggest that BMP4 enhances mitophagy in the lungs affected by bleomycin and acts to inhibit cellular senescence, highlighting its considerable promise as a therapeutic option in the treatment of PF.

The role of the relationship between 8-oxoguanine DNA glycosylase 1 (OGG1), macrophage polarization, and mitophagy in the development of PF has been revealed recently. OGG1, a DNA repair enzyme, not only participates in oxidation-induced DNA damage repair but also aggravates mitochondrial dysfunction by inhibiting PINK1-Parkin mediated mitophagy activation [66]. In PF, macrophage polarization manifests as M1 and M2 types. M2 macrophages activate and proliferate fibroblasts, advancing PF [67]. Elevated OGG1 expression post-BLM-PF correlates with increased M2 macrophages, and TH5487 drug can mitigate OGG1 expression, thereby alleviating PF. Inhibition of PINK1-Parkin mitophagy partially reduces OGG1 inhibition’s protective effect against BLM-PF *in vivo* [68]. These results suggest that OGG1 inhibits mitophagy by negatively regulating PINK1-Parkin expression, thereby promoting M2-type macrophage polarization and the development of PF. Therefore, regulating the activity of OGG1 may become a new strategy to intervene in PF and macrophage polarization.

### Asthma

Asthma, recognized as the most prevalent chronic respiratory condition globally, affects an estimated 320 million individuals [69,70]. Environmental pollutants or allergens such as dust mites can cause allergic asthma [71,72]. Its hallmark features include shortness of breath, wheezing, airflow limitation, and airway hyperresponsiveness [73]. The pathophysiological essence of asthma lies in airway inflammation, which is caused by the overproduction of ROS. Mitochondria, known as a major contributor to ROS, have been observed to show irregularities in the epithelial and smooth muscle cells of individuals with asthma, as well as in mouse models of allergic asthma [74]. Dysfunction of these organelles, coupled with dysregulated mitophagy, results in heightened ROS production and the release of pro-inflammatory cytokines, which are instrumental in the pathogenesis of asthma [73].

Studies have shown that the PINK1-Parkin pathway generally shows an up-regulated trend in asthma. Compared with the healthy control group, the mRNA and protein levels of PINK1 and Parkin in bronchial epithelial cells, lung tissue, and airway fibroblasts of asthma patients were significantly increased. This phenomenon was further verified in the lung tissue of an asthma mouse model induced by ovalbumin, suggesting that PINK1-Parkin-mediated mitophagy is the core response mechanism of asthma pathophysiology [75]. In addition to the classical pathway, the autophagy receptor BNIP3 is specifically up-regulated in specific cell types: the expression of BNIP3 in airway smooth muscle cells (ASMCs) of asthma patients is significantly increased. The mitophagy mediated by BNIP3 can promote the proliferation and migration of ASMCs and directly participate in the pathological process of airway remodeling in asthma [76,77].

Although mitophagy-related proteins such as PINK1, BNIP3, and FUNDC1 typically exhibit compensatory up-regulation in various pathologies, their expression is significantly reduced in nasal polyp tissues of asthma patients with chronic rhinosinusitis with nasal polyps (CRSwNP) [78]. This paradoxical phenomenon may stem from CRSwNP’s disease heterogeneity and microenvironmental dysregulation. Specifically, tissue hypoxia induces abnormal HIF-1α accumulation [79], which activates the NLRP3 inflammasome to up-regulate IL-17A-exacerbating neutrophilic inflammation and epithelial-mesenchymal transition (EMT) [80,81], thereby amplifying airway inflammation and mitochondrial dysfunction. Concurrently, excessive mtROS accumulation drives compensatory depletion of autophagy-related proteins, synergistically aggravating mitophagy impairment [82].

Targeting the mitophagy pathways can significantly regulate the course of asthma, and its mechanism of action exhibits multi-dimensional complexity. Oxidatively activated calcium-calmodulin-dependent protein kinase II (ox-CaMKII) regulates mitochondria homeostasis through a dual mechanism [83]. On the one hand, ox-CaMKII phosphorylates BECN-1, drives the K63 polyubiquitination activation of the PINK1-Parkin pathway, and recruits the OPTN adaptor protein to mediate the ubiquitination of mitochondria and the binding of LC3-II to clear damaged mitochondria [84]. On the other hand, excessive activation of ox-CaMKII can interfere with the fusion of autophagosomes and lysosomes, resulting in the blockage of the autophagic flow [84]. This, instead, leads to an increase in the accumulation of mitochondrial fragments and the outbreak of ROS. Meanwhile, the epigenetic regulatory network precisely co-ordinates the monitoring of mitochondrial quality: for instance, ESR2-mediated inhibition of miR-423 expression induces mitochondrial membrane permeability transition by relieving the transcriptional repression of PINK1, thereby promoting the release of inflammatory factors such as IL-1β; while the DEK protein stabilizes the ATAD3A-DRP1 complex to accelerate excessive mitochondrial division, thereby amplifying the activation of the NLRP3 inflammasome [85]. These findings collectively indicate that precise intervention at key nodes of mitophagy can effectively break the vicious cycle of oxidative stress and inflammation in asthma.

## Molecular cross-talk: mitophagy at the intersection of inflammation

The interplay between mitophagy and inflammatory signaling forms a regulatory axis that dictates disease progression. Below we dissect key molecular dialogues central to lung pathophysiology.

### Overview

Mitochondria are central to maintaining pulmonary cellular homeostasis, orchestrating AECII cell senescence and apoptosis, modulating macrophage immune responses, and fine-tuning endothelial cell repair mechanisms [86]. Their functional integrity—and the efficiency of mitophagy—directly governs lung health. Under physiological conditions, mitochondria-derived molecules act as signaling mediators, but mitochondrial damage disrupts membrane integrity, triggering prolonged mitochondrial permeability transition pore (mPTP) opening and outer membrane permeabilization. These events release mitochondrial components, such as mtDNA and mtROS, into the cytosol as DAMPs, initiating inflammatory cascades.

Cytosolic mtDNA activates two key pathways: the cGAS-STING axis, where cGAS detects mtDNA to synthesize cyclic GMP-AMP (cGAMP), activating STING and driving type I interferon (IFN) and pro-inflammatory cytokine production [87]; and TLR9 recognition of hypomethylated CpG motifs in mtDNA, which activates NF-κB to up-regulate tumor necrosis factor-alpha (TNF-α), IL-6, and other inflammatory mediators [88]. mtDNA also directly stimulates the NLRP3 inflammasome, promoting caspase-1-dependent maturation of IL-1β and IL-18 [89]. Concurrently, mtROS amplifies inflammation by sustaining MAPK activation, stabilizing HIF-1α, and enhancing NF-κB-mediated transcription of NLRP3 and pro-IL-1β. Together, these mechanisms link mitochondrial dysfunction to inflammatory lung pathology [90].

Mitophagy counteracts this process by selectively removing damaged mitochondria, thereby limiting mtDNA and mtROS release. The PINK1-Parkin pathway is particularly critical, as its activation suppresses NLRP3 inflammasome activity and downstream cytokine release. Intriguingly, inflammation reciprocally regulates mitophagy: NF-κB not only drives pro-inflammatory cytokine production but also up-regulates autophagy receptors like p62/SQSTM1, enhancing mitochondrial clearance. However, inflammasome activation can exacerbate mitochondrial damage via ROS-dependent mtDNA release, creating a feedforward loop that sustains inflammation.

**Figure 3:**
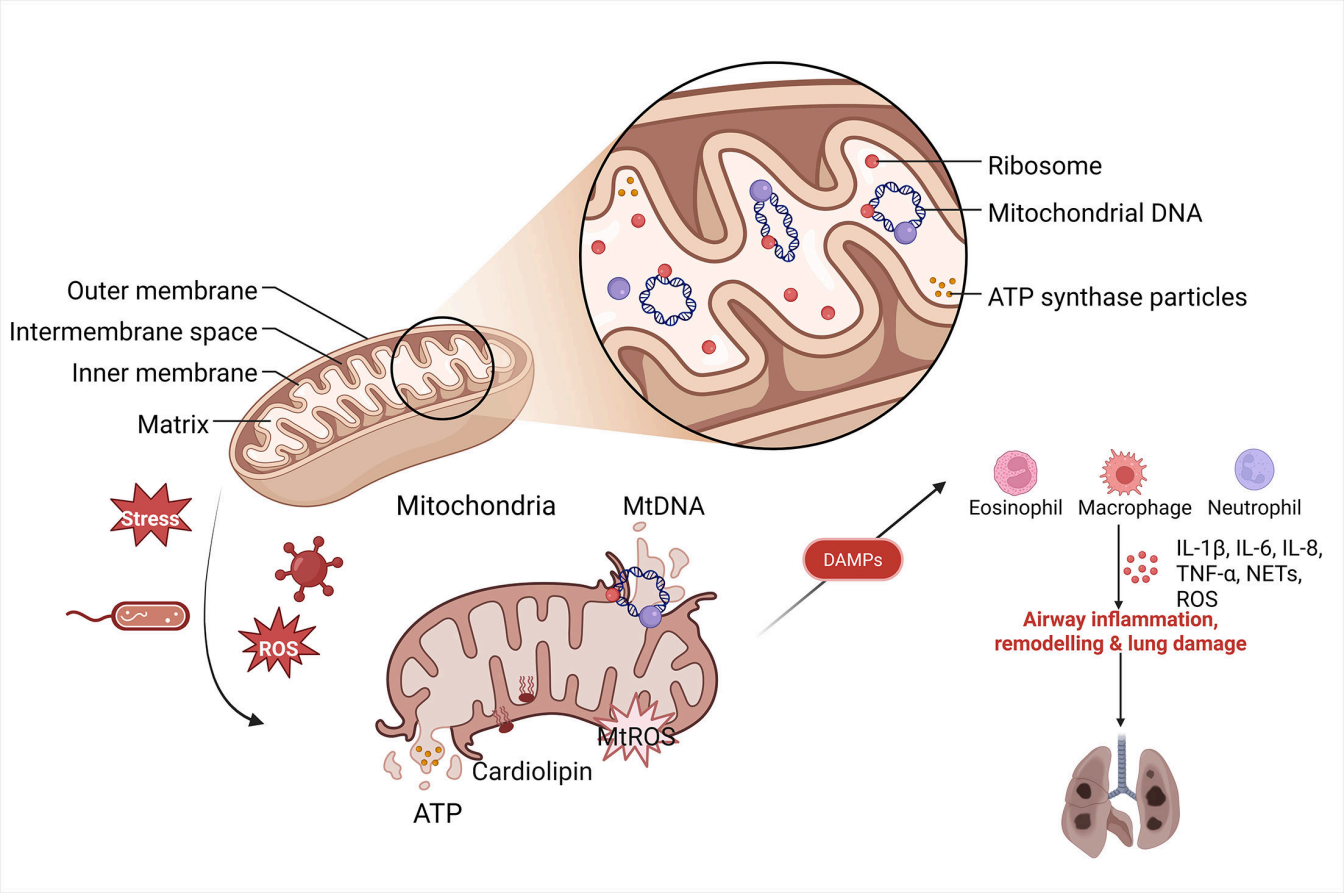
Mitochondria and mtDAMPs. It shows the structure of mitochondria, including the outer membrane, inner membrane, intermembrane space, and matrix. Under stress conditions, mitochondria produce mtDAMPs, such as mtDNA and cardiolipin. These molecules can activate eosinophils, macrophages, and neutrophils, leading to the release of inflammatory factors (such as IL-1β, IL-6, IL-8, TNF-α) and ROS, resulting in airway inflammation, remodeling, and lung injury. mtDAMPs, mitochondrial damage-associated molecular patterns; mtDNA, mitochondrial DNA; IL, interleukins; ROS, reactive oxygen species; TNF-α, tumor necrosis factor-alpha. Created in Biorender.u0. (2025) https://BioRender.com/673z9qy.

This bidirectional interplay raises a pivotal question: Can balancing inflammatory signaling and mitophagy enhance immune defense while preventing pathological inflammation? Current research emphasizes mitophagy’s role in suppressing inflammation, yet the reciprocal regulation—how inflammatory pathways modulate mitochondrial quality control—remains underexplored. Unraveling these mechanisms is essential for understanding lung diseases and broader inflammatory disorders. The following analysis delves into the molecular cross-talk between inflammatory mediators and mitophagy, offering insights into therapeutic strategies that target this dynamic axis (Figure 3).

### Mitophagy and NLRP3 inflammasome

**Figure 4:**
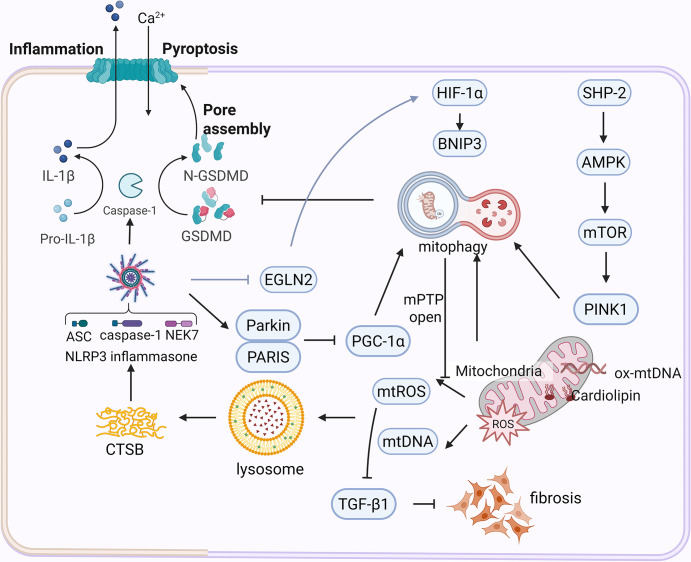
Cross-talk of NLRP3 inflammasome and mitophagy. Upon mitochondrial damage, the elevated mtROS activates the NLRP3 inflammasome, triggering pyroptosis and inflammatory responses. Concurrently, NLRP3 suppresses mitophagy through Parkin degradation, HIF-1α inhibition, and PARIS stabilization, leading to mPTP opening and mitochondrial dysfunction. Conversely, mitophagy eliminates damaged mitochondria via the PINK1-Parkin pathway, reducing mtROS production and NLRP3 activation, thereby mitigating inflammation and pyroptosis. Under hypoxic conditions, HIF-1α induces BNIP3-mediated mitophagy to attenuate mtROS and NLRP3 activation, whereas under stress conditions, mitophagy paradoxically activates NLRP3, promoting inflammatory responses and fibrosis. AMPK, activating AMP-dependent protein kinase; BNIP3, BCL2/Adenovirus E1B 19 kDa protein-interacting protein 3; HIF-1α, hypoxia-inducible factor-1α; GSDMD, gasdermin D; mTOR, mammalian target of rapamycin; mPTP, mitochondrial permeability transition pore; mtROS, mitochondrial reactive oxygen species; NLRP3, NOD-like receptor thermal protein domain associated protein 3; PINK1, PTEN-induced putative kinase 1. Created in Biorender, u0. (2025) https://BioRender.com/1rsdiui.

The inflammasome-mitochondrial axis plays a pivotal role in maintaining immune homeostasis, with its dysregulation driving pathological inflammation through bidirectional cross-talk. Under physiological conditions, mitophagy—mediated by the PINK1-Parkin and BNIP3 pathways—preserves mitochondrial integrity by eliminating damaged organelles, thereby suppressing mtROS accumulation and NLRP3 inflammasome activation. Conversely, defective mitophagy disrupts this equilibrium, triggering a self-amplifying cascade: impaired clearance of dysfunctional mitochondria promotes mtROS overproduction and mPTP opening, which activates NLRP3 [91]. This activation further exacerbates mitochondrial damage through caspase-1-dependent cleavage of Parkin, inhibiting mitophagy and creating a pathogenic feedback loop (Figure 4).

When mitochondria sustain damage, there is a surge in mtROS, which increases the permeability of the lysosomal membrane and facilitates the release of cathepsin B into the cytoplasm. This release triggers the activation of the NLRP3 inflammasome, leading to pyroptosis [92]. On the flip side, the PINK1-Parkin pathway of mitophagy curbs ROS production, inhibits the activation of NLRP3, and diminishes the inflammatory mediators IL-1β and IL-18, thus controlling pyroptosis [93]. Furthermore, mitophagy also plays a role in regulating the opening of the mPTP. Defective mitophagy fails to effectively remove damaged mitochondria, leading to mPTP opening, mitochondrial swelling and rupture, and apoptosis [94]. MPTP opening also leads to increased ROS, further exacerbating oxidative damage and creating a vicious cycle that promotes frequent mPTP opening. The absence of PINK1 further increases ROS release, activating NLRP3-associated pyroptosis [94].

Hypoxia exemplifies adaptive mitophagy regulation, where HIF-1α induction enhances BNIP3-mediated mitochondrial turnover, reducing mtROS and NLRP3 activation while attenuating fibrotic pathways like TGF-β1 [95]. Paradoxically, under stressors such as ozone exposure, ozone-induced mitochondrial dysfunction co-activates the PINK1-Parkin pathway and NLRP3, culminating in caspase-1-mediated processing of pro-IL-1β, IL-18, and gasdermin D (GSDMD), thereby executing pyroptosis and amplifying inflammation [96].

NLRP3 inflammasome further modulates mitophagy via multiple mechanisms: caspase-1-driven Parkin degradation impairs PINK1-Parkin signaling, evidenced by reduced LC3B lipidation and mtDNA accumulation [97], NLRP3 stabilizes EGL-9 family hypoxia-inducible factor 2 (EGLN2) to degrade HIF-1α, suppressing BNIP3 expression [95], and NLRP3 stabilizes PARIS, a Parkin-interacting protein that represses peroxisome proliferator-activated receptor gamma coactivator 1α (PGC-1α)-dependent mitochondrial biogenesis [98]. NLRP3 deficiency reverses these effects, enhancing mitophagy and mitochondrial regeneration. Therapeutic strategies targeting NLRP3 inhibition, PARIS degradation, or ROS scavenging show promise in restoring mitochondrial quality control and interrupting inflammatory cascades, particularly in hyperoxia-induced lung injury and fibrosis. This bidirectional regulatory network underscores the interdependence of mitochondrial homeostasis and inflammasome activity, offering multi-target approaches for inflammatory diseases.

### Mitophagy and cGAS-STING pathway

**Figure 5:**
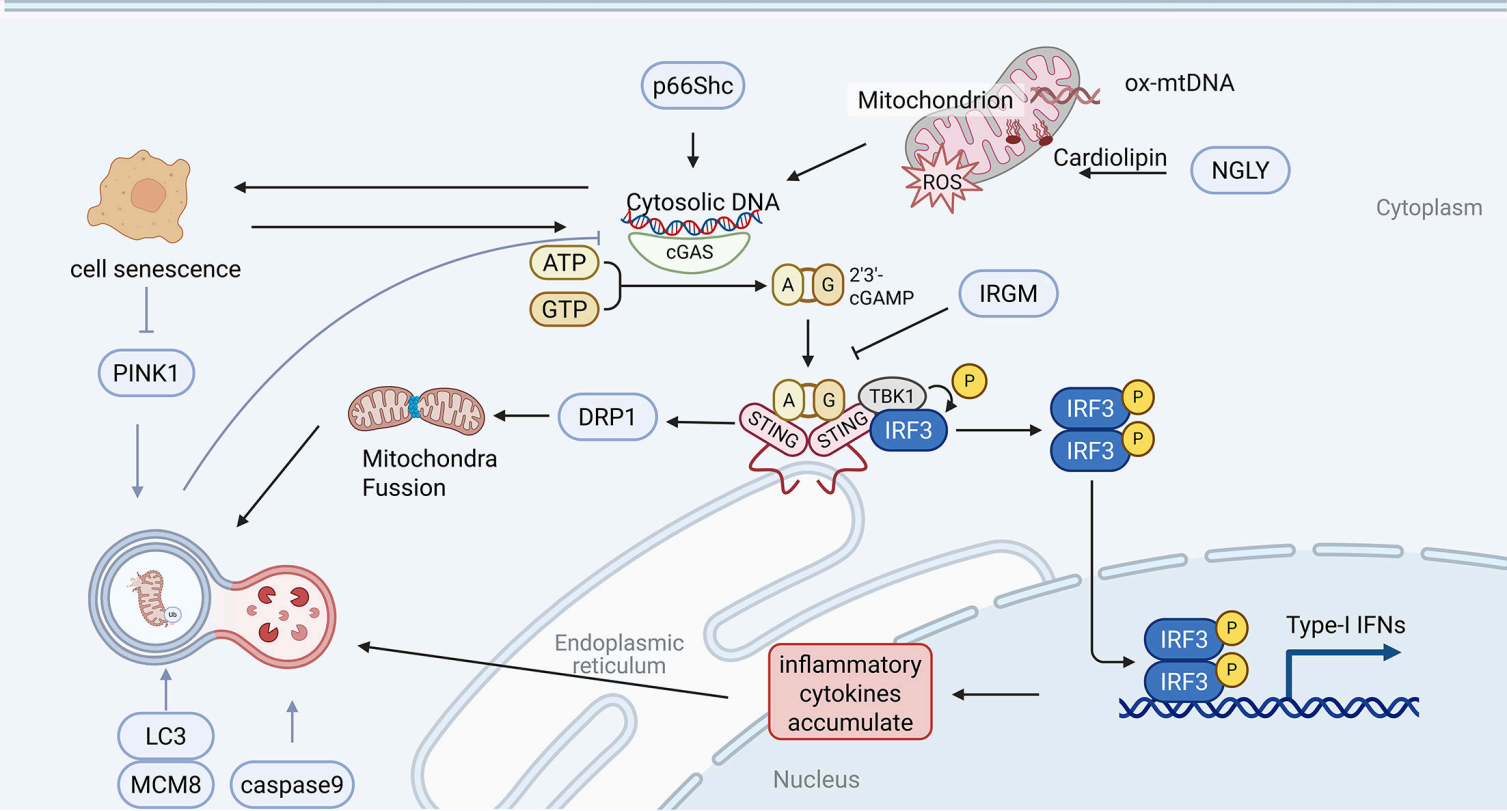
Cross-talk of cGAS-STING and mitophagy. When mitochondria are damaged, they release oxidized mtDNA into the cytosol, activating the cGAS-STING pathway. cGAS detects this cytosolic DNA and produces 2'3'-cGAMP, which binds to STING, leading to the activation of IRF3 and subsequent production of type I IFNs and inflammatory cytokines. Simultaneously, STING promotes mitochondrial fission by up-regulating DRP1, segregating damaged mitochondria for mitophagy. Mitophagy, driven by proteins like PINK1 and MCM8, clears these damaged mitochondria, preventing further mtDNA leakage and dampening cGAS-STING activation. cGAS, cyclic GMP-AMP synthase; cGAMP, cyclic GMP-AMP; STING, stimulator of interferon genes; DRP1, dynamin-related protein 1; IFN, interferon; IRF3, interferon regulatory factor 3; MCM8, minichromosome maintenance 8 homologous recombination repair factor. Created in Biorender, u0. (2025) https://BioRender.com/rhy6w9j.

The cGAS-STING pathway is a key part of the immune response, in which the essential cGAS proteins and STING proteins have unique structural features that are critical to their function [99]. CGAS has a unique dual structural domain, including a nucleotidylyl transferase structural domain and a DNA-binding structural domain, which allows it to sense aberrant free DNA [100]. When DNA fragments escape from the nucleus and mitochondria into the cytoplasm under conditions such as cellular stress or DNA damage, they are recognized by cGAS to synthesize cGAMP [101]. Then cGAMP binds to STING, a transmembrane protein featuring a crucial C-terminal structural domain found in the ER, resulting in a conformational shift of STING that prompts STING to move to the Golgi apparatus [102]. This sequence of events subsequently draws in TANK-binding kinase 1 (TBK1) and inhibitor of kappa B kinase (IKK), leading to their activation, which in turn activates nuclear translocation of interferon regulatory factor 3 (IRF3) and NF-κB to initiate IFN and pro-inflammatory cytokine production. While essential for host defense, dysregulated cGAS-STING signaling drives pathological inflammation [99,103,104] (Figure 5)

The cGAS-STING pathway has emerged as a key sensor and effector in the release of DAMPs, including mtDNA and mtROS. STING activation promotes mitochondrial fission by up-regulating DRP1 while suppressing fusion proteins MFN1/2, segregating damaged mitochondria for mitophagy clearance [105]. The reduced ability to fuse mitochondria can lead to the segregation of impaired ones, stopping them from merging with their healthier relatives. This paves the way for the start and progression of mitophagy. In the end, the cGAS-STING pathway can affect lysosomal pH and the activity of enzymes, ultimately influencing how effectively autophagy materials are broken down. Conversely, impaired mitophagy in aging or disease allows mtDNA accumulation, hyperactivating cGAS-STING and creating a pathogenic feedback loop. In aging macrophages, diminished PINK1-Parkin-mediated mitophagy fails to clear damaged mitochondria, exacerbating cytosolic mtDNA leakage and STING-driven inflammation. Restoring mitophagy—via PINK1 overexpression or TFEB-dependent lysosomal activation (such as Torin-1 treatment)—reduces mtDNA release and suppresses cGAS-STING signaling, highlighting mitophagy’s role in mitigating age-related inflammation [106–108].

The cGAS-STING pathway further intersects with mitophagy through regulatory proteins. Immunity-related GTPase M inhibited the activation of the cGAS-STING and RIG-I-MAVS signaling axes by promoting the autophagy degradation of cGAS and RIG-I and reduced the production of IFN [109], while minichromosome maintenance 8 homologous recombination repair factor suppresses mtDNA-induced STING activation by recruiting LC3 to mitochondria for selective clearance [110]. Intriguingly, STING can operate independently of cGAS, as shown in models where mtDNA release activates STING-IRF3 signaling without cGAS involvement. For instance, BCO2 deficiency induces mtROS-driven mitochondrial fission and mtDNA leakage, activating STING independently to exacerbate inflammation. These findings underscore the pathway’s adaptability: while cGAS and STING typically co-operate in pathogen sensing, their functional divergence enables context-specific immune regulation.

Apart from its role in aseptic inflammatory diseases, cGAS is also involved in immune responses in lung diseases caused by bacterial infections. Wang’s study reported that cGAS-deficient mice exhibited a heavier bacterial load and lung injury after infection with *Pseudomonas aeruginosa*, accompanied by an excessive inflammatory response [111]. Loss of cGAS brings about accumulation of mtDNA in the cytoplasm, which in turn hyperactivates inflammasome and TLR9 signaling. In addition, cGAS deficiency also affected the mitophagy process, resulting in increased mtDNA release. mtDNA in the cytoplasm in turn activates cGAS—a cycle reversed by restoring mtDNA degradation [111]. This reciprocal regulation between mitophagy and cGAS-STING underscores their interdependence: mitophagy limits inflammation by clearing immunogenic mtDNA, whereas cGAS-STING activation can enhance mitochondrial turnover under stress. Such dynamic cross-talk positions the cGAS-STING-mitophagy axis as a therapeutic target for inflammatory diseases, balancing immune defense with mitochondrial homeostasis.

### Mitophagy and NF-κB pathway

**Figure 6:**
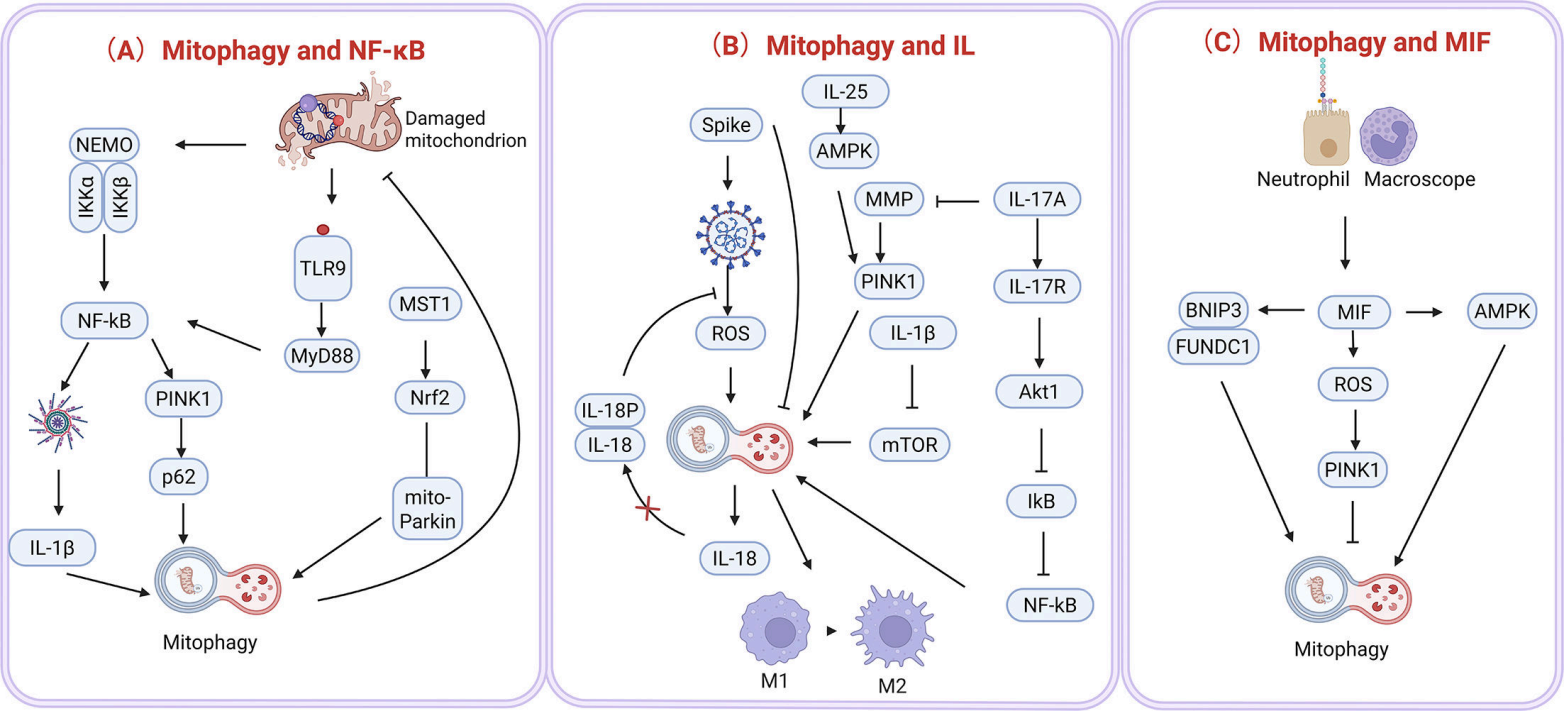
Cross-talk of NF-κB, IL, MIF, and mitophagy. (A) Mitophagy and NF-κB: Damaged mitochondria activate TLR9, which then activates NF-κB through the MyD88 signaling pathway. The activation of NF-κB promotes the expression of PINK1, which further binds to p62, triggering mitophagy. Additionally, damaged mitochondria also activate NRF2 and MST1, promoting the occurrence of mitophagy. The activation of NF-κB also leads to the production of IL-1β, further enhancing the inflammatory response. (B) Mitophagy and IL: The viral Spike protein activates PINK1 through the AMPK and MMP signaling pathways, thereby promoting mitophagy. The activation of PINK1 also leads to the production of IL-1β, which further activates IL-18 and IL-18P through the mTOR signaling pathway. IL-17A activates NF-κB through the IL-17R and Akt1 signaling pathways, thereby promoting the inflammatory response. Additionally, IL-25 activates PINK1 through the AMPK signaling pathway, further promoting mitophagy. (C) Mitophagy and MIF: Neutrophils and macrophages activate AMPK through the MIF signaling pathway, thereby promoting the expression of PINK1 and triggering mitophagy. MIF further promotes the occurrence of mitophagy through the BNIP3 and FUNDCl signaling pathways. The production of ROS also activates PINK1, promoting mitophagy. MIF, migration inhibitory factor; MST1, macrophage stimulating 1; MyD88, myeloid differentiation primary response protein 88; NF-κB, nuclear factor-κB; NRF2, nuclear factor erythroid 2-related factor 2; TLR9, toll-like receptor 9. Created in Biorender.u0, (2025) https://BioRender.com/3fqromg.

The NF-κB pathway orchestrates inflammatory responses to diverse stimuli, including immune challenges and cellular stress, while also modulating mitochondrial quality control through bidirectional interactions with mitophagy. Activation of NF-κB hinges on phosphorylation of the IKK complex, which triggers downstream signaling that up-regulates the autophagy receptor p62. This receptor directs damaged mitochondria for mitophagy degradation, thereby limiting NLRP3 inflammasome activation and IL-1β production. Through ‘NF-κB-p62-mitophagy’ axis, NF-κB paradoxically suppresses its own pro-inflammatory activity, establishing a self-regulatory loop that balances immune activation with tissue repair [112,113] (Figure 6A).

Central to this interplay is NEMO, a regulatory component of the IKK complex. During mitochondrial stress, Parkin recruits NEMO to damaged mitochondria, where it undergoes polyubiquitination and activates NF-κB to amplify inflammatory cytokine production. Simultaneously, NEMO collaborates with p62 to promote mitophagy, ensuring the removal of dysfunctional mitochondria while fine-tuning NF-κB-driven inflammation. This dual role positions NEMO as a critical integrator of mitochondrial quality control and inflammatory signaling, enabling cells to adapt to oxidative stress [114].

The macrophage-stimulating 1 (MST1) kinase further links mitophagy to NF-κB regulation. MST1 typically impedes Parkin translocation to mitochondria, suppressing mitophagy. Its inhibition enhances Parkin-mediated mitochondrial clearance and activates nuclear factor erythroid 2-related factor 2 (NRF2), a redox-sensitive transcription factor that antagonizes NF-κB signaling by reducing extracellular matrix degradation. Thus, MST1 suppression simultaneously boosts mitophagy and dampens inflammation, highlighting its potential as a therapeutic target in oxidative stress-related pathologies [115].

PINK1-mediated mitophagy also intersects with NF-κB through mtDNA dynamics. In lung endothelial injury models, PINK1 silencing reduces mitophagy and mtDNA release, attenuating TLR9-myeloid differentiation primary response protein 88 (MyD88)-NF-κB pathway activation and subsequent inflammation. Conversely, PINK1 overexpression exacerbates mtDNA-driven TLR9 signaling, amplifying NF-κB activity and cellular damage. These findings underscore mtDNA’s role as a DAMP that bridges mitophagy efficiency to inflammatory outcomes, offering strategies to modulate the TLR9-NF-κB axis in conditions like chronic lung injury [88].

Collectively, these mechanisms reveal NF-κB’s dual role as both an initiator and suppressor of inflammation, contingent on its interplay with mitophagy. By coupling mitochondrial quality control to inflammatory signaling, cells dynamically balance damage resolution with immune activation, a paradigm critical for maintaining tissue homeostasis.

### Mitophagy and IL family

Interleukins (ILs) represent a diverse group of cytokines that are produced by various cells and interact with them as well. They are important in maintaining immune system balance and homeostasis. To date, at least 38 ILs have been identified, ranging from IL-1 to IL-38. Members of this family exhibit a broad spectrum of biological functions, including the stimulation of cell growth and differentiation, boosting anti-infective and cancer-fighting responses, driving inflammatory reactions, and influencing cellular metabolism [116,117]. The IL family is organized into specific categories based on their structural and functional properties, which include groups such as IL-1, IL-2, IL-6, IL-10, IL-12, and IL-17, all of which are integral to immune response regulation [118]. Interestingly, recent research has highlighted that certain ILs, like IL-6, IL-18, IL-17A, and IL-25, also play a role in regulating mitophagy (Figure 6B).

Within the IL-1 family, cytokines such as IL-18 and IL-1β play crucial roles in the regulation of mitophagy. IL-18, a proinflammatory cytokine primarily secreted by monocytes and macrophages, has been implicated in COVID-19-related cardiopulmonary injury. In murine models, Spike protein-induced mitochondrial dysfunction—marked by impaired mitophagy and elevated ROS—activated IL-18 in cardiomyocytes and lung endothelial cells. Therapeutic blockade of IL-18 binding to its inhibitor IL-18BP mitigated lung inflammation, underscoring mitophagy and ROS as potential therapeutic targets [119]. Similarly, IL-1β, a key mediator of NLRP3 inflammasome activity, modulates mitophagy through mTOR inhibition, facilitating the clearance of damaged mitochondria in macrophages [120].

IL-6, whose levels rise with aging due to vascular smooth muscle cell activation and mitochondrial instability, contributes to atherosclerosis progression. Age-associated mitochondrial dysfunction reduces mitophagy efficiency, leading to Parkin accumulation and further IL-6 up-regulation, thereby perpetuating a pathogenic cycle [121].

Within the IL-1 family, cytokines such as IL-18 and IL-1β play crucial roles in the regulation of mitophagy. IL-18, a proinflammatory cytokine primarily secreted by monocytes and macrophages, has been implicated in COVID-19-related cardiopulmonary injury. In murine models, Spike protein-induced mitochondrial dysfunction—marked by impaired mitophagy and elevated ROS—activated IL-18 in cardiomyocytes and lung endothelial cells. Therapeutic blockade of IL-18 binding to its inhibitor IL-18BP mitigated lung inflammation, underscoring mitophagy and ROS as potential therapeutic targets [119]. Similarly, IL-1β, a key mediator of NLRP3 inflammasome activity, modulates mitophagy through mTOR inhibition, facilitating the clearance of damaged mitochondria in macrophages [120]. IL-6, whose levels rise with aging due to vascular smooth muscle cell activation and mitochondrial instability, contributes to atherosclerosis progression. Age-associated mitochondrial dysfunction reduces mitophagy efficiency, leading to Parkin accumulation and further IL-6 up-regulation, thereby perpetuating a pathogenic cycle [121].

IL-17A, a hallmark cytokine of Th17 responses, induces mitochondrial depolarization and autophagosome formation in fibroblast-like synoviocytes, as observed in rheumatoid arthritis [116,122]. In fluoride-exposed murine models, elevated IL-17A levels activate the IL-17R-Act1 axis, suppressing IκB in the NF-κB pathway. This cascade promotes mitochondrial damage and mitophagy, evidenced by reduced ΔΨm and altered expression of PINK1, Parkin, and LC3-II in bronchial fibroblasts. Another IL-17 family member, IL-25 (IL-17 E), regulates macrophage polarization via mitophagy. By stimulating ROS production in THP-1-derived macrophages, IL-25 activates AMPK, up-regulates PINK1-Parkin, and enhances LC3-II conversion. This process drives M2 macrophage polarization, linking mitophagy to anti-inflammatory responses and Th2 immunity [123–126].

The IL-12–15-18 cytokine triad impairs mitophagy in natural killer (NK) cells by down-regulating OPA1, a mitochondrial fusion protein. Reduced cristae density and dysfunctional mitophagy contribute to mitochondrial remodeling in cytokine-induced memory-like NK cells, ultimately compromising their viability. Separately, macrophage migration inhibitory factor (MIF), a proinflammatory cofactor of NLRP3, antagonizes mitophagy in sepsis-associated acute kidney injury (SA-AKI). While LPS up-regulates both MIF and PINK1, excessive MIF disrupts Parkin recruitment to mitochondria, inhibiting mitophagy initiation. Pharmacological MIF inhibition restores Parkin-mediated mitophagy, improving renal outcomes in SA-AKI models [127–131] (Figure 6C).

These findings establish ILs as pivotal regulators of mitophagy, with their dysregulation contributing to inflammatory and degenerative diseases. Targeting IL-mediated mitophagy pathways holds therapeutic promise, though further mechanistic studies are needed to dissect context-dependent roles and optimize intervention strategies.

## Therapeutic targeting of mitophagy in lung diseases

Several genetic methods targeting components of the mitophagy mechanism, as a cutting-edge exploration in the field of precision medicine, have shown significant potential in alleviating toxic damage caused by the accumulation of dysfunctional mitochondria in various disease models. This section particularly focuses on the application of genetic and pharmacological strategies in pulmonary diseases, highlighting the broad prospects of mitophagy regulation in maintaining lung health and therapeutic interventions.

### Genetic modulation

Genetic modulation of mitophagy regulators has emerged as a transformative strategy in pulmonary medicine, offering precision tools to address mitochondrial dysfunction in diseases ranging from ALI to COPD. By targeting key mitophagy components—including PINK1, Parkin, SIRT family proteins, and FUNDC1—researchers are unraveling novel therapeutic avenues to restore mitochondrial homeostasis and mitigate inflammatory pathology.

The PINK1-Parkin axis remains central to mitophagy regulation. In PF models, lentiviral-mediated PINK1 overexpression counteracts oxidative stress and mitochondrial dysfunction in lung fibroblasts, while its silencing exacerbates hyperoxia susceptibility in endothelial cells. Complementary studies in hyperoxia-induced lung injury demonstrate that PINK1 synergizes with NLRP3 deficiency to enhance mitochondrial biogenesis and proteasomal clearance via Parkin-PARIS-PGC-1α interactions. Similarly, Parkin overexpression in heat stroke models improves AEC survival by restoring mitophagy flux and mitochondrial integrity, whereas Parkin knockout amplifies ROS-driven PDGFR-PI3K-AKT signaling [131–134].

FUNDC1-mediated mitophagy demonstrates organ-specific protective roles. In pulmonary arterial smooth muscle cells, FUNDC1 overexpression enhances mitophagy and ROS production while stabilizing HIF-1α—effects reversed by its knockdown [135]. LPS-induced ARDS models reveal FUNDC1 deficiency exacerbates lung injury through impaired mitophagy, amplified NLRP3 activation, and antioxidant depletion. These insights underscore FUNDC1’s role as a stress-responsive regulator of pulmonary mitochondrial quality control [136].

The sirtuin (SIRT) family bridges metabolic regulation with mitophagy. SIRT1 activation in diabetic lung transplant models preserves mitochondrial quality through PINK1-mediated mitophagy, while its deficiency accelerates COPD progression by impairing FOXO3 deacetylation and PINK1 expression [137]. SIRT3, a mitochondrial deacetylase, emerges as critical in sepsis-induced lung injury, where its loss disrupts Parkin-mediated mitophagy, fueling NLRP3 inflammasome activation via mtROS and mtDNA leakage. These findings position SIRT proteins as dual modulators of redox balance and inflammatory signaling [138].

Emerging targets further expand the mitophagy toolkit. TFEB overexpression attenuates LPS-induced alveolar damage by up-regulating LAMP1 and autophagy (LC3B, Beclin) markers, while HMGA2 modulation rescues chromium-induced mitochondrial defects through LC3-dependent pathways [139,140]. TROP2, a lung stem cell marker, unexpectedly regulates DRP1-PINK1-driven mitophagy in cigarette smoke models [141]. Conversely, m6A demethylase FTO influences BNIP3 stability in sepsis, linking epitranscriptomic mechanisms to mitochondrial clearance [142].

Collectively, these advances highlight the precision achievable through genetic mitophagy modulation. By tailoring interventions to specific molecular nodes—whether enhancing PINK1-Parkin flux in fibrosis, restoring SIRT3 activity in sepsis, or targeting FUNDC1 in hypoxia—researchers are pioneering strategies to break the cycle of mitochondrial dysfunction and inflammation. The challenge lies in translating these findings into clinically viable therapies that balance mitophagy activation with tissue-specific safety, particularly in aging or comorbid populations. As molecular tools evolve, from viral vectors to RNA-targeting agents, the goal of achieving context-aware mitochondrial precision medicine grows increasingly attainable.

### Pharmacological strategies

The emerging role of mitophagy in respiratory pathologies presents novel therapeutic opportunities. While conventional mitophagy modulators (such as mTOR inhibitors, ROS scavengers) have been extensively characterized, this section focuses on understudied strategies, including traditional medicine, bioactive compounds, and lifestyle interventions.

#### Traditional Chinese medicine

TCM formulations are increasingly recognized for their ability to modulate mitophagy and mitochondrial function, offering novel therapeutic strategies for inflammatory lung diseases.

Bufei Jianpi formula (BJF), a herbal formulation, demonstrates protective effects in COPD models by enhancing mitochondrial biogenesis and function. In rat models of COPD and L6 skeletal muscle cells, BJF improves lung function, mitigates tissue pathology, and restores mitochondrial parameters—including ΔΨm, respiratory capacity, and ATP production—through activation of the AMPK signaling pathway. These effects highlight its dual role in counteracting mitochondrial dysfunction while promoting metabolic resilience in pulmonary and musculoskeletal systems [88].

Liang-Ge-San (LGS), another TCM formula, exhibits broad anti-inflammatory properties in ALI models. In LPS-challenged mice and virus-infected RAW264.7 macrophages, LGS reduces pro-inflammatory cytokine release (IL-6, TNF-α, IL-1β) and suppresses α7nAChR-mediated mitophagy. Mechanistic studies reveal that LGS inhibits TNF-α-induced mitochondrial damage and ROS overproduction in AECs (MLE-12), breaking the cycle of autophagy dysregulation and inflammation. In co-culture systems, LGS attenuates Poly(I:C)-triggered autophagy in epithelial cells while reducing macrophage-derived inflammatory mediators. Notably, LGS extends survival in ALI mice infected with SARS-CoV-2, H1N1, or Poly(I:C), underscoring its therapeutic potential in viral and sterile lung injury [143].

Xijiao Dihuang Decoction combined with Yinqiao Powder (XDY) addresses viral pneumonia by targeting mitophagy-ROS-NLRP3 cross-talk [143]. In influenza-infected macrophages, XDY activates selective autophagic clearance of damaged mitochondria, reducing mtROS accumulation and subsequent NLRP3 inflammasome activation. This dual action inhibits pyroptosis—evidenced by decreased GSDMD-N cleavage and IL-1β maturation—and attenuates lung inflammation. Crucially, XDY’s effects are autophagy-dependent, as the inhibitor 3-methyladenine reverses its suppression of the ROS-NLRP3 axis [143]. By integrating mitochondrial quality control with inflammatory pathway regulation, XDY exemplifies a synergistic approach bridging TCM principles and molecular pathophysiology.

These formulations exemplify the convergence of traditional medicine and modern mitophagy research. Their shared ability to resolve mitochondrial stress while dampening inflammation positions TCM as a rich source of multitarget therapies for complex lung pathologies. Future studies should prioritize elucidating bioactive components, optimizing bioavailability, and validating efficacy in clinically relevant models to advance these therapies toward translational application.

#### Natural products

##### Alkaloids and flavonoids

Protopine (PTP) is an alkaloid with anti-inflammatory and antioxidant properties [144]. Studies reveal that PTP suppresses pro-inflammatory chemokine production by inhibiting the MAPK signaling pathway. In LPS-stimulated RAW264.7 macrophages, PTP significantly reduces inflammatory mediator release [145]. Preclinical investigations in cecal ligation and puncture (CLP)-induced sepsis models indicated that PTP treatment not only lowered mortality rates but also attenuated ALI by inhibiting apoptosis, reducing inflammatory cytokines (such as IL-6, TNF-α), and enhancing glutathione levels and superoxide dismutase activity. Notably, PTP modulates mitophagy and prevents excessive autophagy during sepsis, suggesting a dual role in mitochondrial quality control and inflammation resolution [146].

Derived from citrus fruits, Naringin exhibits robust anti-inflammatory and antioxidant effects, coupled with the ability to reduce cell death rates [147]. Mechanistic studies demonstrate that Naringin suppresses ERS-associated gene expression and inhibits downstream ERS protein activation. Concurrently, it up-regulates the transcription factor ATF3 while down-regulating PINK1 transcription, effectively attenuating mitophagy. These actions position Naringin as a promising candidate for IPF therapy, where dysregulated ERS and mitophagy contribute to disease progression [148].

Resveratrol (RSV), a polyphenolic compound renowned for its anti-inflammatory and antioxidant properties, shows protective effects in ALI/ARDS models. In CLP-induced lung injury, RSV reduces inflammatory cell infiltration, interstitial edema, and levels of CRP, IL-6, IL-1β, and TNF-α, highlighting its role in restoring mitochondrial homeostasis during inflammatory crises [149].

##### Terpenoids and lignans

Sesquiterpenes, a class of natural lignans, exhibit anti-asthmatic properties by modulating mitochondrial and immune pathways. In murine models of CRE-induced asthma, sesquiterpenes markedly suppress inflammatory cell infiltration. Mechanistic studies indicate that these compounds attenuate TH2-driven immune responses by inhibiting TH2 cell activity and down-regulating PINK1 and Parkin expression. Furthermore, sesquiterpenes reduce TNF-α-IL-4-induced mtROS levels, enhance ΔΨm, and inhibit apoptosis in lung airway epithelial cells. Crucially, sesquiterpenes block the translocation of PINK1 and Parkin to mitochondria, suggesting a dual role in preserving mitochondrial integrity and suppressing excessive mitophagy [150].

Gypenoside XLIX (Gyp-XLIX), recognized for its anti-inflammatory efficacy, demonstrates protective effects in sepsis-associated lung injury. Study has demonstrated that Gyp-XLIX pretreatment reduced pathological lung injury and inflammatory factor expression in murine models [149]. This compound inhibits ROS generation and NLRP3 inflammasome activation, likely mediated through the SIRT1-NRF2 signaling axis. Notably, Gyp-XLIX counteracts sepsis-induced apoptosis and mitigates hyperactivation of the PINK1-Parkin mitophagy pathway, highlighting its capacity to restore mitochondrial homeostasis [149].

Kahweol, a diterpene derived from coffee beans, mitigates sepsis-associated ALI by enhancing mitochondrial dynamics and promoting mitophagy through CaMKII-AMPK signaling [151], which not only improves mitochondrial homeostasis but also reduces inflammatory responses and oxidative damage. Preclinical studies reveal that kahweol preserves alveolar structure, attenuates cytokine storms, and stabilizes mitochondrial networks in septic models. Its ability to balance mitophagy while suppressing pathological inflammation positions kahweol as a promising candidate for sepsis-induced ALI therapy [151].

##### Phenolic derivatives

Polydatin (PD), a bioactive stilbenoid glycoside derived from Thuja orientalis and other medicinal plants, demonstrates therapeutic potential in sepsis and burn-associated ALI. Its protective effects are mediated through the preservation of mitochondrial integrity via Parkin-dependent mitophagy activation. In ARDS models, PD significantly attenuates mitochondrial apoptosis and alveolar damage by enhancing the clearance of depolarized mitochondria, thereby disrupting the cycle of caspase-3 activation and oxidative stress amplification. This mechanism positions PD as a promising candidate for mitigating organ failure in critical care settings [152–154].

##### Bioactive hormones and metabolites

Melatonin emerges as a multifaceted therapeutic agent in ALI, exerting antioxidant and anti-inflammatory effects through mitophagy regulation. In sepsis-induced ALI models, melatonin counteracts OPTN suppression to restore mitophagic flux while inhibiting STAT3-mediated epithelial barrier disruption. Its efficacy extends to modulating mitochondrial dynamics via SIRT3-dependent deacetylation of SOD2, reducing oxidative stress and apoptosis in LPS-challenged lung epithelia. Crucially, melatonin’s protective effects are mediated selectively through MTNR1B receptor activation, as demonstrated by receptor-specific inhibitors and genetic knockdown, positioning it as a targeted therapy for ALI [130,155,156].

Epoxyeicosatrienoic acids (EETs), endogenous lipid mediators derived from arachidonic acid, mitigate alveolar epithelial senescence by balancing NOX4-NRF2 redox signaling. EETs suppress excessive mitophagy while enhancing cellular resilience, offering a novel approach to combat age-related pulmonary decline through metabolic pathway modulation [157–159].

##### Nutritional interventions

Study has revealed that vitamin K2 significantly ameliorated histopathological changes in ALI, attenuating inflammation, apoptosis, and tight junction damage, as well as increasing antioxidant enzyme activity, decreasing Ca²^+^ overload, modulating mitochondrial function, and inhibiting pulmonary autophagy in lung tissue [160]. These findings suggest that vitamin K2 may have potential applications in the treatment of ALI [160].

In addition to vitamin K2, vitamin D3 has been found to attenuate lung inflammation. Chen’s group found that vitamin D3 pretreatment reduced intercellular cell adhesion molecule 1 (ICAM-1) expression and leukocyte adhesion, as well as reducing the accumulation of mtROS and inhibiting expression of proteins involved in mitochondrial fission and mitophagy [161], in TNF-α-treated A549 cells. These effects were associated with the regulation of mitochondrial fission and mitophagy via the AKT and NF-κB pathways, suggesting that vitamin D3 has the ability to reduce the expression of adhesion molecules in models of airway inflammation, which may provide a theoretical basis for its use as a novel therapeutic agent targeting epithelial activation of inflammation in the lung [161].

##### Emerging modulators

Nitrated fatty acid (NFA) has anti-inflammatory and antioxidant effects. It was also found that 10-nitrooleic acid was able to reverse the expression of mitophagy-related markers (PINK1 and SQSTM1/p62) induced by hyperoxia, and by regulating mitophagy, NFA helps to restore the mitochondrial homeostasis and protects lung tissues from hyperoxia-induced injury [162].

A study has found that hydrogen ameliorates cellular damage in inflammatory states and promotes mitophagy, and this process is dependent on PINK1 activity. In RAW 264.7 macrophages, the beneficial effects of hydrogen on mitophagy, as well as against cellular injury, were eliminated by knockdown of PINK1. Furthermore, in a CLP mouse model, hydrogen effectively inhibited ALI by activating PINK1-mediated mitophagy [163].

##### Lifestyle interventions

Disease remission and treatment cannot be based on medication alone; a balanced, healthy lifestyle will make a difference in recovery. In most cases, increased mitochondrial biogenesis is beneficial, and in skeletal muscle, caloric restriction enhances mitochondrial function by promoting mitochondrial protein turnover [164]. High-intensity interval training increases the expression of SIRT1, nuclear PGC-1α, and mitochondrial transcription factor A, which increases skeletal muscle mitochondrial numbers and improves exercise performance [165]. Thus, appropriate exercise, such as aerobic exercise, may help to increase lung muscle strength and endurance while promoting mitochondrial biogenesis and autophagy processes in the lungs [166]. In addition, calorie restriction through dietary modification may help activate SIRT1 and PGC-1α, which in turn promotes the health and functional recovery of lung mitochondria [167].

##### Challenges in clinical translation

Tissue-specific mitophagy regulation remains a key hurdle. Alveolar versus bronchial epithelia exhibit divergent responses to PINK1 modulation, necessitating cell-selective delivery systems. Off-target effects of antioxidants (such as NAC) may inadvertently suppress stress-adapted mitophagy. Furthermore, age-related declines in TFEB and PGC-1α impair lysosomal-mitochondrial cross-talk, limiting therapeutic efficacy in elderly patients. Combinatorial regimens (such as exercise-induced SIRT1 activation paired with Parkin enhancers) may overcome these barriers.

This integrative approach—spanning pharmacological agents, dietary factors, and lifestyle interventions—underscores the potential of mitophagy-centric therapies to redefine pulmonary disease management. Success will hinge on resolving delivery precision, temporal control, and age-specific pathway modulation to transform mechanistic insights into clinically viable solutions.

However, the translation of mitophagy modulators into clinical applications is confronted with some formidable challenges. First, patient heterogeneity leads to therapeutic uncertainty. Pulmonary diseases, such as COPD, exhibit significant clinical subtype variations, such as emphysematous and chronic bronchitis, each with distinct pathological mechanisms and molecular profiles [168–170]. The expression levels of key mitophagy proteins (such as PINK1, Parkin, or FUNDC1) in lung tissues of patients with different types of diseases remain unclear. Moreover, genetic background, disease stage, and comorbidities further influence pathway baseline activity, resulting in varied patient responses to the same intervention. Second, existing pharmacological modulators possess inherent limitations. Despite the reporting of various mitophagy modulators in preclinical studies, their clinical translation faces fundamental constraints: the pathway is tightly coupled with autophagy/apoptosis/inflammation, and small molecules often perturb multiple pathways simultaneously, leading to unforeseen toxicities (such as excessive activation causing depletion of healthy mitochondria) [171,172]. Most compounds struggle to achieve effective concentrations in lung tissue and have short half-lives requiring frequent dosing. Systemic modulation of fundamental physiological processes may disrupt energy metabolism homeostasis, and potential carcinogenic risks remain unassessed. Therefore, the identification and development of more effective mitophagy modulators is urgently needed [170,171,173].

## Conclusion and outlook

Mitophagy has emerged as a pivotal process in pulmonary pathophysiology, demonstrating dual roles as both a guardian of cellular homeostasis and a modulator of inflammatory responses in lung diseases. This review synthesizes recent advances in understanding how mitophagy intersects with inflammatory pathways across diverse conditions—from ALI and COPD to asthma and PF. Key insights reveal mitophagy’s context-dependent nature: while its activation typically mitigates oxidative stress and dampens NLRP3-cGAS-STING-driven inflammation, dysregulation can paradoxically fuel pathological cascades through unresolved mitochondrial damage. Therapeutic strategies targeting specific regulatory axes—such as PINK1-Parkin in ALI/ARDS or FUNDC1 in hypoxic fibrosis—show promise in preclinical models, supported by pharmacological agents ranging from natural compounds (melatonin, resveratrol) to TCM formulations. These interventions highlight the potential to recalibrate mitochondrial quality control while preserving physiological function.

Despite progress, critical gaps hinder clinical translation. Current evidence predominantly relies on rodent models and immortalized cell lines, which inadequately capture the complexity of human disease heterogeneity. Most existing therapies lack tissue specificity, raising concerns about off-target effects on non-pulmonary systems. Furthermore, the interplay between aging, comorbidities (e.g., diabetes), and mitophagy remains underexplored, particularly regarding age-related declines in lysosomal function that may compromise therapeutic efficacy.

To bridge these gaps, future research should prioritize three fronts. First, developing precision tools—such as alveolar macrophage-targeted Parkin enhancers—to achieve cell type-specific mitophagy modulation. Second, integrating multi-omics approaches (spatial transcriptomics, metabolomics) with dynamic mitophagy flux assays to map disease-specific regulatory networks in human lungs. Third, advancing combinatorial therapies that pair mitophagy modulators with anti-inflammatories (NLRP3 inhibitors) or mitochondrial biogenesis agonists (PGC-1α activators) to address multifactorial pathology. Clinical validation of traditional formulations like Xijiao Dihuang Decoction through rigorous trials will be essential to assess safety and efficacy in diverse populations.

The evolving landscape of mitophagy research underscores its potential as a therapeutic linchpin in respiratory medicine. By elucidating its nuanced interactions with inflammatory pathways and addressing current limitations, the field may unlock precision strategies for intractable conditions like IPF and severe asthma. Success will require balancing mechanistic innovation with translational rigor, ultimately transforming mitochondrial homeostasis from a biological concept into a cornerstone of clinical care.

## Open Access

This article has been published open access under our Subscribe to Open programme, made possible through the support of our subscribing institutions, learn more here: https://portlandpress.com/pages/open_access_options_and_prices#conditional

## Abbreviations

AEC: alveolar epithelial cell
AECII: alveolar epithelial type II
ALI/ARDS: acute lung injury/acute respiratory distress syndrome
AMPK: AMP-dependent protein kinase
ASMCs: airway smooth muscle cells
ATP: adenosine triphosphate
BCAP31: B-cell receptor-associasted protein 31
BECN-1: Beclin-1
BJF: Bufei Jianpi formula
BNIP3: BCL2/Adenovirus E1B 19 kDa protein-interacting protein 3
CCCP: carbonyl cyanide 3-chlorophenylhydrazone
CERS1: ceramide synthase 1
CIC: citrate carrier
CL: cardiolipin
CLP: cecal ligation and puncture
COPD: chronic obstructive pulmonary disease
CRSwNP: chronic rhinosinusitis with nasal polyps
CS: cigarette smoke
CSE: cigarette smoke extract
DRP1: dynamin-related protein 1
EETs: epoxyeicosatrienoic acids
EGLN2: EGL-9 family hypoxia-inducible factor 2
EMT: epithelial-mesenchymal transition
ER-Mito: endoplasmic reticulum and mitochondria
FUNDC1: FUN14 domain-contacting protein 1
GSDMD: gasdermin D
Gyp-XLIX: Gypenoside XLIX
HIF-1α: hypoxia-inducible factor-1α
ICAM-1: intercellular cell adhesion molecule 1
IFN: interferon
IKK: inhibitor of kappa B kinase
ILs: interleuklins
IMM: inner mitochondrial membrane
IPF: idiopathic pulmonary fibrosis
IPF: idiopathic pulmonary fibrosis
IRF3: interferon regulatory factor 3
LAMP1: lysosomal associated membrane protein 1
LC3: microtubule-associated protein 1 light chain 3
LGS: Liang-Ge-San
LPS: lipopolysaccharide
MAPK: mitogen-activated protein kinases
MERC: mitochondria-ER contact
MFN1/2: mitofusin1/2
MIF: macrophage migration inhibitory factor
MPP: mitochondria-penetrating peptide
MST1: macrophage-stimulating 1
MUL1: mitochondrial E3 ubiquitin protein ligase 1
MyD88: myeloid differentiation primary response protein 88
NDP52: nuclear dot protein 52 kDa
NFA: nitrated fatty acid
NF-κB: nuclear factor-κB
NK: natural killer
NLRP3: NOD-like receptor thermal protein domain associated protein 3
NM23-H4: hexameric membrane spacer protein NDPK-D
OGG1: 8-oxoguanine DNA glycosylase 1
OMM: outer mitochondrial membrane
OPA1: optic atrophy 1
OPTN: optineurin
PARL: progerin-associated rhodopsin
PD: polydatin
PF: pulmonary fibrosis
PGC-1α: proliferator-activated receptor gamma coactivator 1α
PINK1: PTEN-induced putative kinase 1
PTP: protopine
ROS: reactive oxygen species
RSV: resveratrol
SA-AKI: sepsis-associated acute kidney injury
SIAH1: seven in absentia homolog-1
SIRT: sirtuin
SIRT3: silent information regulator 3
SQSTM1/p62: sequestosome-1
STING: stimulator of interferon genes
Sens2: sestrin2
TBK1: TANK-binding kinase 1
TCM: traditional Chinese medicine
TIM: translocase of inner mitochondrial membrane
TLR9: toll-like receptor 9
TNF-α: tumor necrosis factor-alpha
TREM-1: triggering receptor expressed on myeloid cells 1
Tβ4: thymosin β4
XDY: Xijiao Dihuang Decoction combined with Yinqiao Powder
cGAMP: cyclic GMP-AMP
cGAS: cyclic GMP-AMP synthase
mPTP: mitochondrial permeability transition pore
mTOR: mammalian target of rapamycin
mTORC1: mechanistic target of rapamycin complex 1
mtDAMPs: mitochondrial damage-associated molecular patterns
mtDNA: mitochondrial DNA
mtROS: mitochondrial reactive oxygen species
ox-CaMKII: oxidatively activated calcium-calmodulin-dependent protein kinase II
